# Editorial: Mental health recovery: engaging and empowering people living with mental illness and their families

**DOI:** 10.3389/fpsyt.2025.1633178

**Published:** 2025-06-06

**Authors:** Wei Zhou, Qi Yuan, Hao Yao, Micheal Lee, Liang Zhou

**Affiliations:** ^1^ Research Center for Public Health and Social Security, School of Public Administration, Hunan University, Changsha, Hunan, China; ^2^ Research Division, Institute of Mental Health, Singapore, Singapore; ^3^ Shanghai Mental Health Center, Shanghai, China; ^4^ Faculty of Medicine, University of British Columbia, Vancouver, BC, Canada; ^5^ The Affiliated Brain Hospital of Guangzhou Medical University, Guangzhou, China

**Keywords:** mental health recovery, people with lived experience (PWLE), personal recovery, engagement, empowerment, recovery-oriented care

The understanding of mental health recovery has undergone a substantial paradigm shift in the past 40 years, evolving from a clinically-oriented model to a more person-centered approach (1, 2). Patricia Deegan, a researcher and a psychologist with lived experience of schizophrenia, provided a compelling illustration of this paradigm shift. She asserts that recovery is to become “the unique, awesome, never to be repeated human being that we are called to be”, while “…liv(ing), work(ing), and lov(ing) in a community in which one makes a significant contribution” (3). This reframes recovery as a process toward authentic selfhood and meaningful community participation. Building on these perspectives, William Anthony introduced the concept of personal recovery in 1993, defined as “a deeply personal, unique process of changing one’s attitudes, values, feelings, goals, skills, and/or roles” (4). The widely cited CHIME framework-Connectedness, Hope, Identity, Meaning, and Empowerment-further operationalizes the key elements of personal recovery (5), capturing the intrapersonal, relational, and social dimensions of recovery. These developments have significantly shaped the landscape of global mental health, advocating for a shift toward recovery-oriented approaches in both practices and policies, as exemplified by the World Health Organization (WHO)’s endorsement in many of its official publications (6).

The collections in this Research Topic reflect the evolving understanding of mental health recovery in the very recent years. They demonstrate how people with lived experience of mental illness can engage in meaningful living through self-reflection, peer connection, institutional innovation, and attitudinal change. Although these studies vary in research focus and methodological design, they all underscore the notion that recovery is a relational and participatory process enacted not only within the individuals but also through their multifaceted interactions with peers, professionals, and the broader communities.

Zhu and Lyu present a collaborative autoethnography reflecting one author’s lived experience of recovery from depression and its intersectionality with broader social contexts. Drawing from anti-stigma perspectives, the paper provides an in-depth account of how individuals make sense of their mental distress and reclaim their identities through personal reflection and social interaction. It highlights how individuals with mental illness can be active agents of change and first-person experts in their own recovery processes.

To translate such subjective experiences into clinical insights, Wozniak et al. review the application of the Repertory Grid Technique, a tool based on personal construct theory, in assessing how inpatients with various mental disorders construct their sense of self and others. The review reveals uniform patterns such as lower self-esteem and idealization of others among mental health inpatients, along with diagnosis-specific cognitive structures. These patterns offer insights for more tailored therapeutic interventions for recovery.

Two studies in this Research Topic focus on peer support, a growing area of interest in mental health recovery (7). Fan et al. describe how peer interactions within a psychiatric day-care center in China foster a sense of meaning and community, despite a lack of a formal structure. In contrast, Lequin et al. examine the integration of trained Peer Mental Health Practitioners (PMHPs) at a hospital unit caring for patients with psychiatric disorders. Their qualitative study demonstrates that structured peer support benefits not only patients but also clinical teams and the institution. However, the authors also note that clearly defined roles and professional boundaries are crucial for the sustainable integration of PMHPs.

Health professionals play a pivotal role in mental health service delivery and recovery processes. Their attitudes significantly influence service users’ self-perception and therapeutic engagement. Evidence suggests that healthcare providers’ attitudes toward mental illness are no more positive than those of the general public (8, 9). Cho and Kim attempted to address this gap by designing an empathy-enhancing program for nursing students, using patient narratives. Their quasi-experimental study shows promising effects of having service users as educators and agents of change through sharing their lived experience narratives with future care providers.

Despite the valuable insights from these studies on engaging and empowering individuals with lived experience, a notable omission remains: the role of families. This omission may also reflect a broader gap in recovery research and practices literature (10). Families often serve as primary caregivers and integral members of interpersonal networks, particularly in non-Western or low-resource settings (11). Engaging families is essential to supporting the individual’s recovery and sustaining their own well-being (12). Future research should prioritize family integration and explore strategies to empower families as collaborative partners in recovery.

In summary, the collections in this Research Topic reaffirm that recovery is neither exclusively a clinical process nor simply an intrapersonal one. Rather, it is relational, context-sensitive, and socially embedded. Meaningful engagement must extend beyond individualistic therapeutic encounters to encompass the broader domains of social relationships, institutional practices, and public narratives. Recovery takes place when people with mental illness become active participants in mental health practices and policies. To enable such recovery, mental health systems must foster recovery-oriented environments both within care settings and across broader society that value inclusion, shared decision-making, the lived experiences of individuals and families, and collective transformation (13, 14).
